# Physical Activity and Academic Procrastination among Chinese University Students: A Parallel Mediation Model of Self-Control and Self-Efficacy

**DOI:** 10.3390/ijerph19106017

**Published:** 2022-05-15

**Authors:** Changqing Li, Yanbo Hu, Kai Ren

**Affiliations:** 1 College of Physical Education and Health Science, Chongqing Normal University, Chongqing 401331, China; 20170005@cqnu.edu.cn; 2 Department of psychology, London Metropolitan University, London N7 8DB, UK; y.hu@londonmet.ac.uk; 3 College of Physical Education and Health Sciences, Zhejiang Normal University, Jinhua 321004, China

**Keywords:** physical activity, self-control, self-efficacy, academic procrastination, parallel mediation model

## Abstract

Previous studies have suggested that physical activity may decrease academic procrastination; however, few studies have explored the underlying mechanisms of how physical activity exerts an effect on academic procrastination. This study aimed to examine the mediating effects of self-control and self-efficacy in the relationship between physical activity and academic procrastination among Chinese university students. Methods: A cross-sectional design was used in this study. The sample comprised 564 university students from a university in Zhejiang, China. The physical activity rating scale-3 (PARS-3), self-control scale (SCS), generalized self-efficacy scale (GSES), and procrastination assessment scale-students (PASS) were used to investigate university students’ physical activity, self-control, self-efficacy, and academic procrastination respectively. The Percentile-Bootstrap technique was performed to examine the mediating effects of self-control and self-efficacy on the association between physical activity and academic procrastination. Results: Physical activity significantly predicted higher levels of self-control and self-efficacy, as well as lower levels of academic procrastination. Self-control and self-efficacy were significant mediators between physical activity and academic procrastination. Conclusion: This study indicated that physical activity interventions targeting the improvement of self-control and self-efficacy may reduce academic procrastination in university students.

## 1. Introduction

Procrastination is considered to be a tendency to voluntarily postpone an intended action despite being aware of the negative consequences [1]. As a specific type of procrastination, academic procrastination refers to postponing the start and completion of study-related tasks and activities [2]. Studies have found that academic procrastination is widespread among university students, with 70–95% reporting such behavior [1]. It has been suggested that academic procrastination is associated with poor academic performance [3] and higher levels of emotional problems, such as stress, depression, and anxiety [4,5,6,7,8].

Therefore, the negative impact and high prevalence rate of academic procrastination necessitated the urgency of exploring methods to decrease academic procrastination. Physical activity, self-control, and self-efficacy were found to be negatively correlated with general procrastination [9,10,11,12,13]. Interestingly, both self-control and self-efficacy were found to be positively associated with physical activity [14,15,16,17,18]. The relationship between these factors has not been established. Therefore, this study investigated whether self-control and self-efficacy mediate the association between physical activity and academic procrastination in university students.

### 1.1. Physical Activity and Procrastination

Physical activity has been found to have a negative relationship with general procrastination [9,12,13]. Individuals who do enough physical activity (more than 150 min per week) were found to be less likely to demonstrate procrastination behavior [9] while less active students suffered higher levels of procrastination [13]. In addition to the duration of physical activity, the intensity level of physical activity also impacts the level of procrastination. Shi et al. tested different levels of physical activity intensities (light, moderate, and high) and found that more intense physical activity leads to less procrastination [12]. Similar findings also been reported by Zhong and Chu [13] who examined the impact of different aspects of physical activity (duration, intensity as well as frequency) on general procrastination amongst 220 Chinese university students.

Although the impact that physical activity exert on general procrastination has been consistent, its effect on academic procrastination in particular is scarce. Therefore, the potential underlying psychological mechanisms between these relationships still need to be further explored.

### 1.2. The Mediating Role of Self-Control

Self-control is defined as the individual’s capacity to override dominant responses, including thoughts, emotions, and actions, in order to reach long-term goals [19,20]. Individuals that scored higher on self-control scales do better in various domains, including health and health associated behaviors [21,22]. Different factors have been found to have an effect on self-control, among which, the level of physical activity was a consistent one. Studies supported that physical activity level could be a predictor of self-control [14,15,17]. Oaten and Cheng tested 24 undergraduate students using a four-month physical exercise program and concluded that physical exercises improved the participants’ level of self-control, which was further related to the performance of their tasks and study [14].

Self-control also plays a crucial role in procrastination [1]. Previous research has attributed procrastination to exhausted self-control resources [23]. On the other hand, people with higher self-control were found to be more able to resist short-term temptations therefore less likely to procrastinate [22,24,25]. Based on the literature reviewed above, it is plausible to presume that physical activity is positively correlated with self-control, which, in turn, is negatively associated with academic procrastination. In other words, self-control might mediate the link between physical activity and academic procrastination.

### 1.3. The Mediating Role of Self-Efficacy

Self-efficacy is defined as an individual’s belief in having required capabilities to complete given tasks [26]. The term of self-efficacy can be seen in specific domains (e.g., academic self-efficacy, exercise self-efficacy, or physical self-efficacy), as well as in a global domain (general self-efficacy) [27]. Most researchers agree that participation in physical activity can have a positive impact on one’s specific self-efficacy. A cross-sectional research showed that the amount of physical activity a person participated in was significantly associated with confidence in his or her ability to exercise regularly, i.e., self-efficacy in exercise [28]. Similarly, McAuley et al. conducted a 6-month exercise trial and found that participants who were more frequently active had more sense of exercise self-efficacy [29]. Furthermore, a variety of physical activities help improve self-efficacy, such as Tai Chi and Yoga [16,18].

On the other hand, it is known that self-efficacy greatly influences individuals’ task initiation and persistence [30]. Low self-efficacy is associated with behavioral avoidance, whereas high self-efficacy promotes behavioral initiation and persistence [26]. Procrastination has been viewed as a type of behavior avoidance [31], so it is possible that low self-efficacy would lead to procrastination. When people do not believe they are capable of completing their assigned tasks, they are less likely to start and persist in completing tasks. Indeed, previous research shows that low level of self-efficacy leads to academic procrastination [10].

Considering the above, it could be expected that physical activity is positively correlated with self-efficacy, which in turn, is negatively associated with academic procrastination. In other words, self-efficacy might mediate the link between physical activity and academic procrastination.

The important role of physical activity in decreasing academic procrastination is not fully recognized so far. Most studies looked into the single relationships between physical activity, self-efficacy, self-control, and academic procrastination. Therefore, the potential underlying psychological mechanisms between these relationships still need to be further explored.

The main goal of this study is to investigate the linkage between physical activity and academic procrastination among university students with self-control and self-efficacy as possible mediators. Based on previous findings in the literature, we hypothesized the following:

**Hypothesis** **1** **(H1).** *Self-control mediated the relationship between physical activity and academic procrastination*.

**Hypothesis** **2** **(H2).** *Self-efficacy mediated the relationship between physical activity and academic procrastination*.

**Hypothesis** **3** **(H3).** *Self-control and self-efficacy parallelly mediated the relationship between physical activity and academic procrastination*.

## 2. Materials and Methods

### 2.1. Participant and Procedure

Full-time and non-athletic university students enrolled in physical education classes (e.g., table tennis, badminton, aerobics) were recruited by convenience sample from a university in Zhejiang, China. There were 564 university students (251 males, 313 females) aged 17–23 years (M = 19.44, SD = 0.87) who completed investigation by the paper-and-pencil questionnaires. The sample consisted of 111 freshmen (19.7%), 362 sophomores (64.2%), and 91 juniors (16.1%). Seniors did not participate in the study as they were not enrolled in any of those physical education classes. 

The data collection conducted in groups in the classroom before participants physical education class began. Paper based questionnaires were distributed. Instructions were explained in detail and all participants signed informed consent form before the study started. All participants were tested on four scales: physical activity, self-control, self-efficacy, and academic procrastination scales. Approximately 8 min was required by each student to complete all items. Demographic information about gender, age, major, and grade were also included in the survey.

#### 2.1.1. Physical Activity

Physical activity was measured using Physical Activity Rating Scale-3 (PARS-3) revised by Liang [32]. The scale included three items in total, focusing on physical activity intensity, physical activity duration, and physical activity frequency. For each item, five choices were listed, each accredited with points ranging from 1 to 5.

For example, the question for physical frequency was how often do you do physical activity every month/week? The corresponding choices were: less than 1 time/month (1 point), 2 to 3 times/month (2 points), 1 to 2 times/week (3 points), 3 to 5 times/week (4 points), and every day (5 points).

The calculation of the overall physical activity score is based on the following equation: physical activity score = physical activity intensity score × (physical activity duration score − 1) × physical activity frequency score. The physical activity score interval ranged from 0 to 100 points. Depending on the physical activity score, the physical activity level was then divided into low, moderate, and high categories: low ≤ 19 points, 20 ≤ moderate ≤ 42 points, and high ≥ 43 points.

#### 2.1.2. Self-Control 

The Self-Control Scale (SCS) [22] was used to assess self-control. This scale involves five dimensions and 19 items in total. The five dimensions are impulse control (e.g., “I often act without thinking through all the alternatives”), resisting temptation (e.g., “I am good at resisting temptation”), focusing on work or study (e.g., “I have trouble concentrating”), healthy habits (e.g., “I have a hard time breaking bad habits”), and moderation (e.g., “I do certain things that are bad for me, if they are fun”). Items were scored on a 5-point Likert scale. The responses across the 19 items were summated as the total score of self-control. Potential scores of SCS range from 19 to 95, and a higher score indicates the higher level of self-control. In the current study, the Cronbach’s α of this scale was 0.85.

#### 2.1.3. Self-Efficacy

Self-efficacy was examined using a 10-item Generalized Self-Efficacy Scale (GSES) [33]. Participants rated each item (e.g., “I can always manage to solve difficult problems if I try hard enough.”) on a 4-point scale ranging from 1 (not at all true) to 4 (extremely true). A total score of GSES is generated by adding together all 10 items (ranging from 10 to 40), with higher score indicating greater levels of self-efficacy. In the current study, the Cronbach’s α of this scale was 0.85.

#### 2.1.4. Academic Procrastination

To assess participants’ academic procrastination, 18 items derived from Procrastination Assessment Scale—Students (PASS) were used in this study [2]. The scale includes six domains (academic tasks) (i.e., writing a term paper, studying for an exam, keeping up with weekly reading assignments, performing administrative tasks, reading books borrowed from others or library, and performing academic tasks in general) with a total of 18 items (three items for each domain). For each academic task, subjects indicate on a 5-point Likert scale about the extent to which they procrastinate on the task (1 = never; 5 = always), the extent to which procrastination on the task is a problem for them (1 = never; 5 = always), and the extent they want to decrease their procrastination on the task (1 = never; 5 = always). Participants indicated the degree of procrastination and the severity of the problem caused by procrastination in six academic tasks using a 5-point Likert scale, ranging from 1 to 5. A higher score signified a higher level of academic procrastination. In the current study, the Cronbach’s α of this scale was 0.91.

### 2.2. Statistical Analysis

Pearson correlation analysis and linear regression analysis were performed to test the relationships among measured variables. The PROCESS macro (model 4) for SPSS developed by Hayes [34] uses the resampling method of bootstrapping, which gives an estimate of the indirect effect based on 5000 bootstrap samples. Bootstrapping was used as it allows us to avoid Type 1 errors that may arise from non-normal distributions of an indirect effect. In this study, 95% CI was employed, which were deemed significant when the 95% CI did not contain zero. 

## 3. Results

### 3.1. Descriptive Statistics and Bivariate Correlation Analysis

Descriptive statistics and correlations for variables were provided in Table 1. Physical activity was found to be positively and significantly correlated with self-control (r = 0.17, *p* < 0.01) and self-efficacy (r = 0.20, *p* < 0.01) in university students. Likewise, academic procrastination was found to have significant reverse relationships with physical activity (r = −0.17, *p* < 0.01), self-control (r = −0.48, *p* < 0.01), and self-efficacy (r = −0.30, *p* < 0.01). 

### 3.2. Regression Analysis and Parallel Mediation Analysis

**Figure 1:**
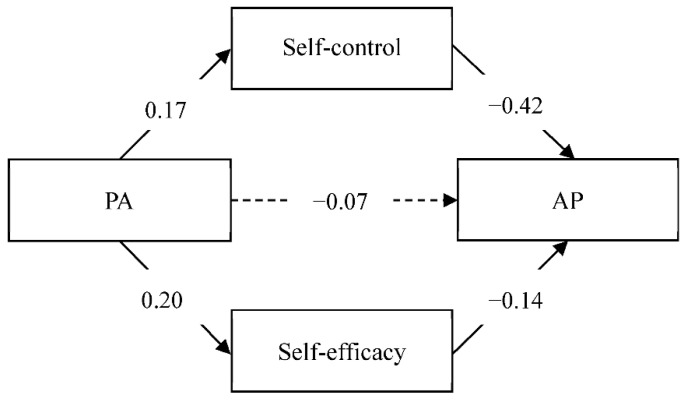
Mediating effect of self-control and self-efficacy on the relationship between physical activity and academic procrastination. PA: physical activity; AP: academic procrastination.

Table 2 and Figure 1 showed the results of the regression analysis. The results indicated that physical activity positively predicted self-control (β = 0.17, *p* < 0.001) and self-efficacy (β = 0.20, *p* < 0.001). In the second step, physical activity, self-control, self-efficacy, and academic procrastination were added to the regression model, it was found that self-control and self-efficacy significantly predicted academic procrastination (β = −0.42, *p* < 0.001, and β = −0.14, *p* < 0.001, respectively), while the relationship between physical activity and academic procrastination was not statistically significant anymore.

The parallel mediation model of the relationship between physical activity and academic procrastination by self-control and self-efficacy was presented in Table 3. The total indirect effect of physical activity on academic procrastination through self-control and self-efficacy was −0.048 (95% CI [−0.069, −0.027]), from which the indirect effects of physical activity on academic procrastination through self-control and self-efficacy were −0.035 (95% CI [−0.054, −0.017]) and −0.013 (95% CI [−0.024, −0.005]), respectively. These confidence intervals suggested that there were significant indirect effects between physical activity and academic procrastination through both mediators. 

## 4. Discussion

This study investigated whether physical activity may impact academic procrastination among university students through self-control and self-efficacy using a parallel mediation model. Consistent with previous studies, our study found that participating in physical activity was found to be positively associated with self-control and self-efficacy [14,15,16,17,18] while negatively associated with academic procrastination among university students [9,12,13]. Self-control and self-efficacy were observed to negatively correlate with academic procrastination as shown in previous studies [10,11]. Furthermore, our results demonstrated that physical activity exert its effect on academic procrastination via self-control and self-efficacy as mediators. 

### 4.1. Physical Activity, Self-Control, and Academic Procrastination

According to strength model, self-control resources work in similar ways to how muscles work. Just like muscle energy can be temporarily worn and then recover or become stronger after appropriate rest, self-control level could also wither by constant use and then recover after resting [25,35]. Tasks with appropriate challenge levels have been suggested to further improve self-control levels [24,35]. Indeed, this has been observed in the process of physical activity training (both aerobic and strength training), if individuals could overcome difficulties and persevere to the end, their self-control level were also strengthened [14,36]. 

In addition, our data also demonstrated that individuals with low level self-control were more prone to academic procrastination as in previous studies [11,37]. This was because lack of self-control resources often leads to being more distracted and easily tempted by irrelevant chores, which delay completion of tasks [37]. 

The strength model could explain how physical activity increased the self-control level. Once self-control level is elevated, more resources are available to deal with the challenges of tasks to complete, which results in less distractions and less academic procrastination. These explains the underlying mechanisms of the mediator effect of self-control we found. 

### 4.2. Physical Activity, Self-Efficacy, and Academic Procrastination

One of the reasons that ours and previous studies [16,18] found a positive relationship between physical activity and self-efficacy was that physical activity provided meaningful mastery experiences [38]. According to social cognitive theory [26], successful experiences were helpful to make individuals feel better about themselves and consequently improve their perceived competence. With regard to physical activity, the stress brought by exercise is predictable and controllable, and therefore more manageable [39]. When individuals repeatedly manage stress successfully, they increase the belief that they could deal with stress in a more positive way. Therefore, physical activity can help improving their sense of mastery and self-efficacy [39]. 

As reported in previous studies, we also found self-efficacy was negatively correlated with academic procrastination [40,41,42]. It is possible that individuals with high levels of self-efficacy are more confident in their abilities, and therefore are more motivated to work harder to achieve their goals, more persistent when confronted with obstacles, and more likely to finish academic tasks on time instead of procrastinating [43]. 

The mediation effect of self-efficacy between physical activity and academic procrastination in this study successfully replicated what we found before [44]. Based on what is discussed above, we believe that physical activity can help students improve their self-efficacy and form the belief that they can successfully cope with obstacles or challenges. The application of this belief in academic settings would help students face challenges with more confidence and therefore reducing academic procrastination.

### 4.3. Limitations 

There are several limitations in the current study that could be addressed in future studies. First, participants could be recruited from more diversified cultures and backgrounds. Currently, all participants are Chinese. Second, causality cannot be inferred from the current study alone and further longitudinal or experimental studies are needed in the future. Third, the dependency on self-report data could potentially pose a risk in validity of the measurements. Last, R^2^ value in this study explained a small portions of variation, and this implied there might be other underlying mediators need to take into account between physical activity and academic procrastination in future studies.

## 5. Conclusions

This study furthers the understanding of the mechanism of how physical activity exerts an effect on academic procrastination. To the best of our knowledge, this is the first study to reveal an indirect relationship between physical activity and academic procrastination, mediated by self-control and self-efficacy in parallel. In light of this, physical activity interventions that focus on improving levels of personal self-control and self-efficacy can help reduce academic procrastination. It is recommendable for university students to engage in moderate or high intensity physical activity regularly to obtain the benefit of physical activity on academic procrastination. Therefore, the importance of physical activity needs to be kept in mind by policy makers and teachers while designing curriculums or activities to promote academic success in university students.

## Figures and Tables

**Table 1:** Descriptive statistics and correlations between variables (N = 564).

Variable	M	SD	1.	2.	3.	4.
1. Physical activity	22.08	16.44	-			
2. Self-control	3.16	0.56	0.17 **	-		
3. Self-efficacy	2.39	0.47	0.20 **	0.35 **	-	
4. Academic procrastination	2.77	0.67	−0.17 **	−0.48 **	−0.30 **	-

** *p* < 0.01.

**Table 2:** Results of regression analysis.

Regression Equation		Fitting Indices		Regression Coefficient	
Outcome Variables	Predictor Variables	R^2^	F	β	t
Self-control		0.03	16.73 ***		
	Physical activity			0.17	4.09 ***
Self-efficacy		0.04	22.52 ***		
	Physical activity			0.20	4.75 ***
AP		0.25	63.03 ***		
	Physical activity			−0.07	−1.80
	Self-control			−0.42	−10.68 ***
	Self-efficacy			−0.14	−3.48 ***

AP: Academic procrastination; *** *p* < 0.001.

**Table 3:** Results of Bootstrap test.

Indirect Effect	Effect Size	Boot SE	Boot 95% CI	
			Lower Limit	Upper Limit
Total	−0.048	0.011	−0.069	−0.027
Self-control	−0.035	0.010	−0.054	−0.017
Self-efficacy	−0.013	0.005	−0.024	−0.005

Boot SE: Boot Standard Error; Bootstrap 95% CI: Bootstrap 95% Confidence Interval.

## Data Availability

The data in the study are not publicly available in order to protect privacy of the participants.
